# Pontocerebellar Hypoplasia Maps to Chromosome 7q11.23: An Autopsy Case Report of a Novel Genetic Variant

**DOI:** 10.1155/2019/7048537

**Published:** 2019-12-10

**Authors:** Kritika Krishnamurthy, Amilcar A. Castellano-Sanchez, Christopher A. Febres-Aldana, Jyotsna Kochiyil, Carole Brathwaite, Robert J. Poppiti

**Affiliations:** ^1^A. M. Rywlin MD Department of Pathology, Mount Sinai Medical Center, Miami Beach, FL, USA; ^2^Florida International University, Herbert Wertheim College of Medicine, Miami, FL, USA; ^3^Department of Radiology, Mount Sinai Medical Center, Miami Beach, FL, USA; ^4^Department of Pathology, Nicklaus Children's Hospital, Miami, FL, USA

## Abstract

Pontocerebellar hypoplasias are a group of autosomal recessive neurodevelopmetal disorders with varied phenotypic presentations and extensive genetic mutational landscape that are currently classified into ten subtypes. This classification is based predominantly on the genetic iterations as the phenotypic presentations are often broad and overlapping. Pontocerebellar hypoplasia type-3 (PCH3) is an autosomal recessive disorder characterized by a small cerebellar vermis, hyperreflexia, and seizures, described in Middle Eastern families in association with a homozygous truncating mutation of the *PCLO* gene in locus 7q11-21. This is a case of PCH, with previously unreported novel genetic alterations. The patient is a 1-week-old girl, born at term to a 26-year-old G4P0A3 woman in a nonconsanguinous relation. At birth, the baby was depressed and hypertonic with abnormal tonic-clonic movements of extremities. MRI revealed cerebellar and brainstem hypoplasia. Postmortem examination revealed a palmar simian crease. The cerebellum measured 2.5 cm from side to side and 1 cm from rostral to caudal. The vermis was rudimentary. Sectioning revealed a flattened linear fourth ventricle, scant abortive cerebellar foliae, and a markedly small cerebellum when compared with the cerebrum and with age-matched size. H&E-stained sections of cerebellum revealed scant rudimentary foliae. A rudimentary unilateral embolliform nucleus was identified. The remaining cerebellar nuclei were absent. Chromosomal microarray showed an interstitial duplication of 841 kB on chromosome 7q11.23. Locus 7q11.23 contains FGL2 and GSAP genes and is 5 MB upstream of the 7q11-21 region, suggesting a possible linkage. This novel genomic finding possibly represents a new familial variant of PCH closely associated with PCH-3 and further strengthens its association with the 7q11 locus.

## 1. Introduction

Pontocerebellar hypoplasias (PCHs) are a genetically and clinically heterogeneous group of neurodevelopmental disorders with autosomal recessive transmission. In the past decade, whole-exome sequencing (WES) has led to the identification of new genes, allowing the recognition of at least 10 different PCH types with broad and overlapping phenotypes [1]. Pontocerebellar hypoplasia type-3 (PCH3), also known as cerebellar atrophy with progressive microcephaly (CLAM) is a rare form characterized by hypotonia and impaired swallowing in the neonatal period and by seizures, optic atrophy, and short stature from infancy onwards, but these clinical findings are nonspecific [2]. This entity has been described in Middle Eastern families in association with a homozygous truncating mutation of the *PCLO* gene in locus 7q11-21 [3, 4]. Herein, we report a case of PCH with a novel genetic variation in the same DNA segment as PCH3.

## 2. Case Presentation

**Figure 1:**
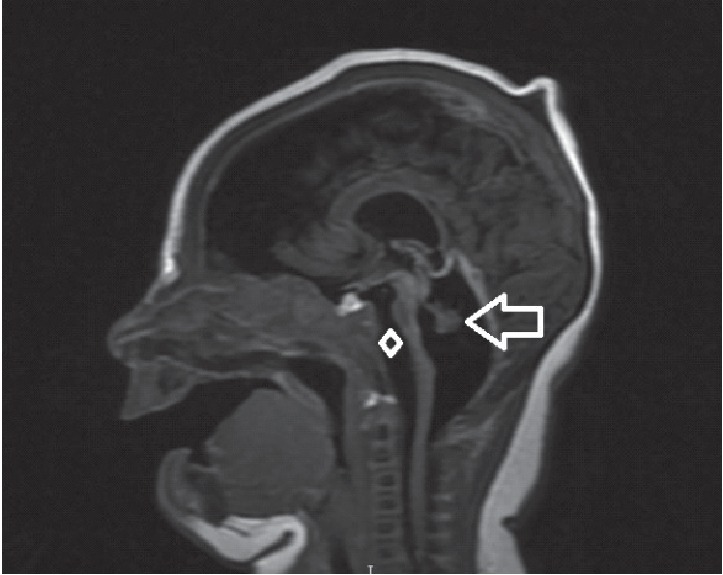
T1 sagittal image showing near complete absence of cerebellum with rudimentary anterior vermis (arrow).There is also marked hypoplasia of the pons also seen in the image (diamond).

The patient is a 1-week-old Hispanic baby girl, born at a gestational age of 38 weeks and 5 days, via cesarean section indicated due to polyhydramnios, meconium-stained fluid, and failure of progression of labor, to a 26-year-old G4P0A3 clinically normal woman in a nonconsanguinous marraige with a clinically normal man. The apgar scores were 5 at one minute, 6 at 5 minutes, and 7 at 10 minutes. The baby had an abnormal fetal ultrasound since 29 weeks of pregnancy showing a small fetal head with intracranial anatomy with possible third ventricle enlargement vs. cavum vergae. At birth, the baby was depressed, hypertonic with contracture of all four limbs. Abnormal tonic-clonic movements of extremities were noted since birth for which the patient was started on phenobarbitone. MRI revealed microcephaly with significant cerebellar and brainstem hypoplasia (Figure 1). An opthalmic and hearing evaluation was planned but not performed due to the poor prognosis. The parents accepted natural death after seven days in the NICU on CPAP.

On postmortem examination, the patient weighed 3200 grams (>50^th^ percentile [5]) and the crown-heel length was 50 cm. Gross examination revealed mild microcephaly with a head circumference of 31.8 cms (<50^th^ percentile [5]) and a simian crease. No facial abnormalities or dysmorphism were noted. Gross and microscopic examination of the cardiovascular, respiratory, gastrointestinal, and genitourinay system failed to reveal any abnormalities.

**Figure 2:**
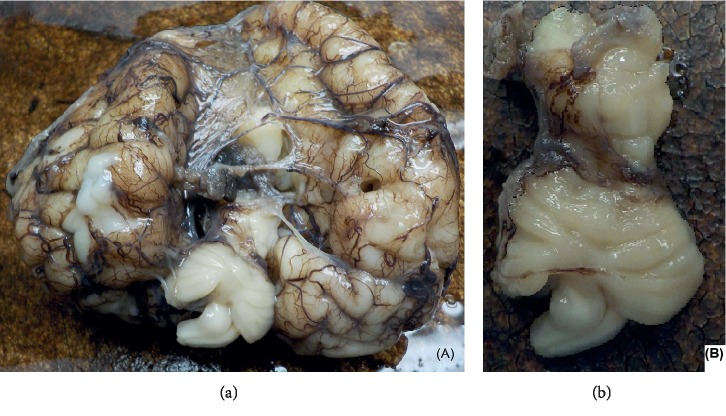
(a) Normal for gestational age cerebrum with markedly diminished cerebellar volume. (b) Marked hypoplastic cerebellum with rudimentary vermis.

The brain weighed 194 grams which was low for the gestational age. The two cerebellar hemispheres were joined by a middle portion that most likely represented the joining vermis and appeared very rudimentary. The pons and medulla also appeared to be rudimentary. The cerebellum in total measured only 2.5 cm from side to side and 1 cm from rostral to caudal, in comparison with the cerebrum, where each hemisphere measured 8 × 6.5 × 5 cm which was adequate for the gestational age (Figure 2). Cerebral hemispheres were cornally sectioned to reveal an ill-defined white to gray matter junction which was more prominent towards the temporal and occipital lobes. The ventricular system was dilated, particularly towards the frontal pole. The brainstem and cerebellum were sectioned to reveal a tan color, the presence of an aqueduct of Sylvius, a flattened linear fourth ventricle within the cerebellar folia, and marked decrease in the parenchyma of the cerebellum. No distinction of the vermis, the foliae, or other cerebellar structures was noted grossly and no clear delineation of the dentate nucleus was observed. The optic nerve was grossly unremarkable.

**Figure 3:**
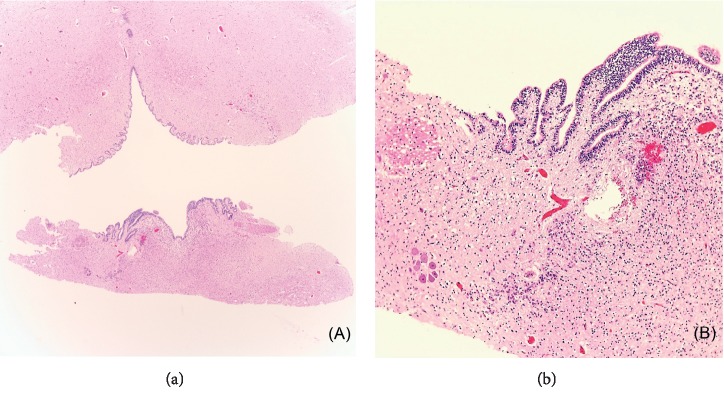
(a) Flattened linear fourth ventricle with scant, abortive foliae and no delineation of cerebellar nuclei. (b) Scant, abortive foliae with molecular, Purkinje, and granular layers.

**Figure 4:**
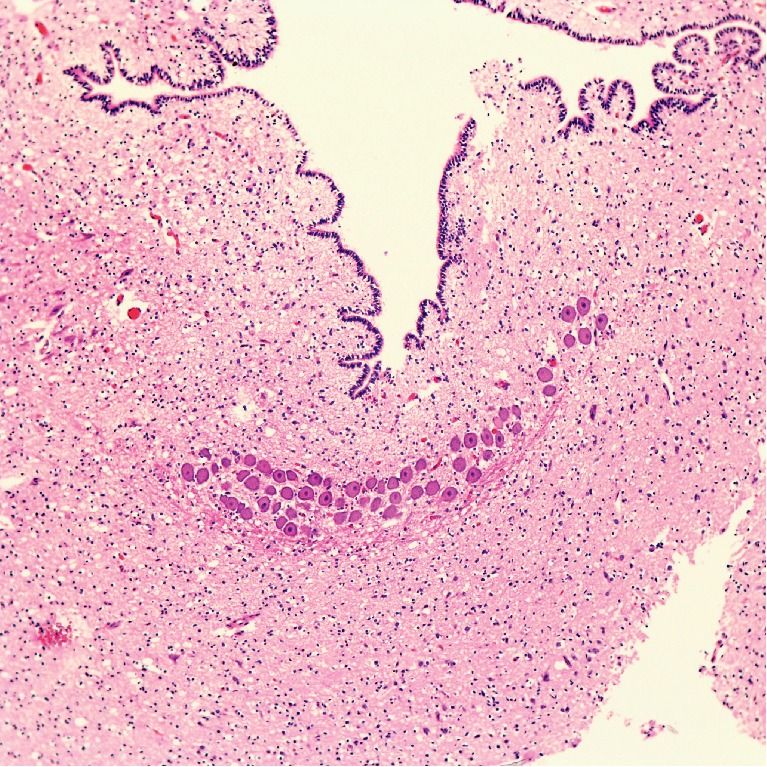
A rudimentary unilateral nucleus.

H&E-stained sections of the brainstem sections from rostral to caudal demonstrated a marked atrophy of the midbrain and a shallow-to-flat pons where only the dorsal pontine nuclei were observed. Sections through the cerebellum revealed a well-formed and large fourth ventricle; however the cerebellar parenchyma only had rudimentary and scanty foliae (Figure 3). The folia showed molecular, Purkinje, and granular layers as well as an external granular layer as expected for the age. A single unilateral rudimentary cerebellar nuclei was seen (Figure 4). This nucleus was thought to most likely represent a rudimentary embolliform nucleus based on the shape and location. Caudally, the medulla oblongata was also atrophic.

Chromosomal microarray was performed at an outside institution after obtaining all appropriate consents from the mother and revealed an interstitial duplication of 841 kB on chromosome 7q11.23. The parents were phenotypically normal and did not have any family history suggestive of underlying genetic disorders and therefore were not tested.

## 3. Discussion

The first description of a patient with a hypoplastic pons and cerebellum can be traced back to the beginning of the 20th century [6]. In 1929, Krause reported the case of a 16-month-old child with swallowing problems, spasticity, and microcephaly who on further investigation was found to have a cerebellum severely diminished in size but grossly normal cortical gyri and sulci [7]. In 1990, Barth described seven cases presenting with microcephaly, spastic paresis, and extrapyramidal dyskinesia in five related families in the Netherlands [8]. Severe pontocerebellar hypoplasia and cerebral atrophy was noted in the CT scans, and histological examination revealed marked loss of neurons in the pons and the cerebellum. Though severe cerebellar and brainstem hypolasia was noted on MRI in our case, the head circumference was only one standard deviation less than the 50^th^ percentile and did not meet the diagnostic criteria for significant microcephaly. [9].

Initially, PCH was classified in two subtypes based on the presence (subtype 1) or absence (subtype 2) of motor neuron degeneration in the anterior horn of the spinal cord. With the advent of next generation sequencing, significant advances have been made over the last two decades in research on pontocerebellar hypoplasia (PCH). Now, based on clinical and genetic features, PCH is classified into ten subtypes. Cerebellar and pontine hypoplasia and/or atrophy, a fetal or early disease onset, and severe developmental delay with extremely limited cognitive and motor skills are the hallmarks shared among all the ten subtypes. The loss of Purkinje cells, fragmentation of the dentate nucleus, and loss of pontine nuclei result in the small volume of the cerebellum and pons. Rudimentary foliation of the cerebellar hemispheres is a common finding [10]. The hypoplastic cerebellar folia along with rudimentary nuclei and hypoplastic brainstem seen in our case are consistent with the diagnosis of PCH.

PCH-3 is a rare subtype described in only a few families of Omani and Middle Eastern pedigree [2, 3]. The symptoms are nonspecific and include progressive microcephaly, seizures during the first year of life, severe developmental delay, truncal hypotonia, increased deep tendon reflexes, and limb spasticity. The abnormal tonic and clonic movements of the extremities seen in our case could represent seizure activity and in conjunction with other findings of mild microcephaly and abnormal body tone may be suggestive of PCH3. However, due to the rarity of PCH in general and PCH3 in particular, it is hard to confirm the subtyping in our case. Facial dysmorphism (low set ears and prominent eyes), short stature, and low weight are also reported. However, no such deformity was noted in our case. In the cases reported thus far, all but one had optic atrophy. Optic atrophy was not present in our case. PCH3 has been described to coexist with tetralogy of Fallot [11] and Vitamin A deficiency [12, 13], but the association with simian crease, as seen in our case, has not been reported previously in the literature.

The etiology of PCH3 remains largely elusive. In 2 families, an implication of locus 7q11-21 has been demonstrated. PCH3 is inherited in an autosomal recessive manner [3]. In our case, the parents were not related to each other and had no family history of genetic defects. As such, the PCH seen in the patient would have required either a sporadic mutation in the germline cells of both parents or a sporadic mutation in the patient's own germ cells during embryogenesis. Recently, a homozygous nonsense mutation in piccolo presynaptic cytomatrix protein (*PCLO*) was identified in an Omani family with PCH3 [4]. *PCLO* is potentially involved in regulation of presynaptic proteins and vesicles [4]. An individual from Turkey was reported to have a remarkably similar clinical presentation and displayed linkage to the chromosomal region encompassing *PCLO*, suggesting possible allelism [2].

This is the first reported case of 7q11.23 associated PCH. The locus 7q11.23 contains *FGL2* and *GSAP* genes and is 5 MB upstream of the 7q11-21 region, which suggests a possible linkage [14]. Heterozygous interstitial 7q11.23 duplication is associated with hypoplasia of cerebellum, corpus callosum, and temporal lobes in children with cognitive impairment meeting the criteria for autism spectrum disorders [15].

Currently, there is no cure for PCH. Treatment is only symptomatic and includes percutaneous endoscopic gastronomy feeding, respiratory support, treatment of dystonia and seizures, and physiotherapy [16]. Age of death ranges from neonatal to late twenties, though most patients die in childhood.

## 4. Conclusions

A homozygous truncating mutation of the *PCLO* gene in locus 7q11-21 has been previously reported in PCH-3. This is the first reported case of 7q11.23 associated PCH. The locus 7q11.23 contains *FGL2* and *GSAP* genes and is 5 MB upstream of the 7q11-21 region. This novel genomic finding represents a new familial variant of PCH and further strengthens its association with the 7q11 locus.
